# Development of a Low-Cost EEG-Controlled Hand Exoskeleton 3D Printed on Textiles

**DOI:** 10.3389/fnins.2021.661569

**Published:** 2021-06-25

**Authors:** Rommel S. Araujo, Camille R. Silva, Severino P. N. Netto, Edgard Morya, Fabricio L. Brasil

**Affiliations:** ^1^Edmond and Lily Safra International Institute of Neuroscience, Santos Dumont Institute, Macaíba, Brazil; ^2^Federal Institute of Education, Science and Technology of Rio Grande Do Norte, Ceara-Mirim Campus, Ceará-Mirim, Brazil

**Keywords:** hand exoskeleton, 3D printing, textiles, soft robotics, post-stroke, rehabilitation, brain-machine interface

## Abstract

Stroke survivors can be affected by motor deficits in the hand. Robotic equipment associated with brain–machine interfaces (BMI) may aid the motor rehabilitation of these patients. BMIs involving orthotic control by motor imagery practices have been successful in restoring stroke patients' movements. However, there is still little acceptance of the robotic devices available, either by patients and clinicians, mainly because of the high costs involved. Motivated by this context, this work aims to design and construct the Hand Exoskeleton for Rehabilitation Objectives (HERO) to recover extension and flexion movements of the fingers. A three-dimensional (3D) printing technique in association with textiles was used to produce a lightweight and wearable device. 3D-printed actuators have also been designed to reduce equipment costs. The actuator transforms the torque of DC motors into linear force transmitted by Bowden cables to move the fingers passively. The exoskeleton was controlled by neuroelectric signal—electroencephalography (EEG). Concept tests were performed to evaluate control performance. A healthy volunteer was submitted to a training session with the exoskeleton, according to the Graz-BCI protocol. Ergonomy was evaluated with a two-dimensional (2D) tracking software and correlation analysis. HERO can be compared to ordinary clothing. The weight over the hand was around 102 g. The participant was able to control the exoskeleton with a classification accuracy of 91.5%. HERO project resulted in a lightweight, simple, portable, ergonomic, and low-cost device. Its use is not restricted to a clinical setting. Thus, users will be able to execute motor training with the HERO at hospitals, rehabilitation clinics, and at home, increasing the rehabilitation intervention time. This may support motor rehabilitation and improve stroke survivors life quality.

## 1. Introduction

Most of the activities of daily living (ADLs) directly involve hand motor skills. However, the occurrence of a stroke can change this reality rapidly, impairing arm and hand function persistently. Stroke is a serious but common disease. In 2017, an estimated 11.9 million people worldwide suffered strokes, representing an increase of 21% in 10 years from 2008 (GBD, 2018), and becoming one of the main causes of long-term disabilities (Benjamin et al., 2018). Six months after the stroke onset, 30% of hemiplegic stroke patients remain with no arm and hand function. This situation is consistent with considerable damages to the corticospinal tract (Kwakkel et al., 2003), which enable the patient to perform basic hand movements such as cylindrical and pinch grasps movements.

One of the primary approaches to recover movement in stroke patients involves medication and active motor training with physiotherapists or occupational therapists (McConnell et al., 2017; Tai et al., 2020). Nonetheless, there are novel approaches that may be included in the treatment to improve the outcomes. A proven effective strategy for motor rehabilitation is the Constraint-Induced Movement Therapy (CIMT), which restricts the movements of a healthy arm/hand in order to reinforce motor learning by the impaired limb, stimulating its use (Wolf et al., 2006; Langhorne et al., 2011; Pollock et al., 2014). Despite its effectiveness, the CIMT fail to engage a large number of patients, mainly due to factors such as the restriction of movement for long periods, the immobilization devices (Page et al., 2002), and the requirement of some residual motor skills in affected limbs (Taub et al., 1999; Wolf et al., 2006). Therefore, severely hemiplegic stroke patients are not able to benefit from this therapy.

Although stroke survivors generally have damaged the cortex or associated neural communication pathways, the ability to perform the mental rehearsal of a physical movement task—know as motor imagery (MI)—remains preserved in most cases (Page et al., 2005; Buch et al., 2008; Bartur et al., 2019; Lu et al., 2020). When a patient performs an MI task with his/her hand, it is possible to identify a sensorimotor rhythm (SMR) desynchronization—also referred to as event-related desynchronization (ERD)—in the motor cortex by processing neuroelectric signals recorded through BMIs (Remsik et al., 2016; Guggisberg et al., 2019).

Recent studies suggest the use of alternative practices such as MI training associated with robotic devices concomitantly with physical therapy (Padfield et al., 2019; Ramos-Murguialday et al., 2019; Bhagat et al., 2020). This approach is considered a promising tool for post-stroke rehabilitation. In the last few years, the advance of new BMI technologies and pioneering results attracted researchers' attention to developing robotic devices to recover hand movements. Electroencephalography (EEG) is a widely used non-invasive BMI system to record brain signals in MI practices due to its high temporal resolution, portability, and relatively low cost (Padfield et al., 2019).

In a broad study (Ramos-Murguialday et al., 2013), 32 chronic stroke patients with paralysis and no extension movement in one hand underwent daily training with an EEG-controlled orthotic device plus behaviorally oriented physiotherapy for 1 month. Patients of the experimental group improved significantly arm and hand movements. A recent study from the same group indicates that significant improvements in upper-limb motor function are partly preserved 6 months after BMI-based rehabilitation (Ramos-Murguialday et al., 2019). Patients were able to control the BMI even with different lesion locations and sizes. In addition, it is important to mention that trying to rehabilitate the hand can improve not only the hand skills but also arm skills (Balasubramanian et al., 2010). For more detailed information regarding MI for upper limb rehabilitation (see Padfield et al., 2019).

### 1.1. Hand Exoskeletons

Exoskeletons for hand rehabilitation had received significant attention from the scientific community due to its ability of being a limb extension (Franceschini et al., 2020). If one project is capable of developing a lightweight, practical, ergonomic, portable, and mainly functional device, one can expect that patients will be more likely to engage in rehabilitation interventions or even take the device to train at home. In this way, stroke patients could leave the clinical setting and increase the therapy intensity. Additionally, the device could also assist the patients in several ADLs.

It is well-known that user requirements, e.g., weight, comfort, esthetics, and cost, are directly related to the success or failure of hand rehabilitation devices (McConnell et al., 2017). Some researchers (Aubin et al., 2013) suggest that the maximum weigh of the exoskeleton over the hand should not exceed 500 g. In a review by Chu and Patterson (2018), all exoskeletons surveyed weighed <285 g over the hand. Actuators and control systems can be attached to the body and should weigh <3 kg, the average weight of portable electronic devices, e.g., laptops (Polygerinos et al., 2015; Haghshenas-Jaryani et al., 2020).

Furthermore, the cost is also one of the major problems faced by designers. High prices may cause patients to seek only conventional treatments, such as medicines and physical therapy. Moreover, the acquisition of such devices may be unfeasible for intensive training within patients' homes. Seeking to solve these problems, several studies have proposed different exoskeletons specifications. They vary as to the total degrees of freedom (DOF), types of mechanisms, and actuators, which may be electric, pneumatic, cable driven, or shape-memory alloys (Chu and Patterson, 2018). Different manufacturing techniques were also applied, with emphasis on three-dimensional (3D) printing (McConnell et al., 2017), a fast, practical, and low-cost prototyping technique, which allows manufacturing of complex structures that could not or would be very expensive, to be produced by other traditional techniques, e.g., machining.

### 1.2. Soft Robotics

Soft Robotics aims to develop ergonomic and biocompatible devices by trying to mimic the biomechanics of living beings. This technology is interesting and suitable to develop new hand exoskeletons because it favors the design of mechanisms with no need to incorporate rigid parts to their projects (Liu et al., 2020). Thus, it is possible to develop more friendly devices with anatomic-like functionalities. In a review by Chu and Patterson (2018), most of the previously developed soft exoskeletons were driven by pneumatic actuators (64%). In sequence were cable-driven devices (34%) and only a small part was driven by hydraulic actuators (2%).

Although pneumatic systems can perform movements similar to natural movements of the hand (Chu and Patterson, 2018), the pumps to compress the air are usually bulky and noisy. Therefore, they end up compromising portability and daily live use (Heo et al., 2012). Even though current devices may be quieter, pneumatic systems are not capable to generate great efforts. Hydraulic actuators follow the same principle, however, the working fluid is a liquid (Polygerinos et al., 2015) and, thus, they are usually heavier than other actuators (Chu and Patterson, 2018).

In cable-driven exoskeletons, the transmission of force generated by the actuators is accomplished by Bowden cables. Usually made of up steel, they are commonly found in automotive and aeronautical industries. These cables have the ability to both pull and push mechanical components even though the path between the actuator and the component is curved. The main advantage of using Bowden cables to drive exoskeletons is because this configuration allows placing the actuators in a separate module, reducing the weight over the hand (Buch et al., 2008; Borboni et al., 2016; Nycz et al., 2016).

### 1.3. 3D Printing

3D printing technology has evolved significantly over the years. At first, 3D printers were used to rapidly prototype initial models to test and adjust specific design details. Currently, it is already possible to find functional 3D-printed devices aiming to lower costs and speed up product customization. Combining soft robotics to 3D printing can, thus, be a solution to the main problems of developing a rehabilitation device for the hand (McConnell et al., 2017).

One of the most widespread, versatile, and accessible 3D printing techniques is the fused deposition modeling (FDM), in which a material—typically a polymer—is fused and extruded onto a surface layer by layer. The molten material of a new layer adheres to the previous layers. We observed that this technique permits one to pause printing to insert porous textiles and then resume printing to form a “fabric sandwich.” Thus, the new layers of molten material are free to adhere to other layers through the pores, enabling the fabrication of wearable devices.

The authors are not aware of any study applying this technique to manufacture hand exoskeletons. Searches on specialized and social media only show applications for entertainment and fashion, e.g., cosplay costumes. In academics, as far as we are aware, only two studies published in proceedings quantitatively analyzed 3D printings with embedded textiles (Sabantina et al., 2015; Rivera et al., 2017). According to Sabantina et al. (2015), PLA filaments have greater adhesion strength. Both the width of the mesh opening and the thickness of the fabrics directly influence the adhesion of the PLA.

Addressing these challenges, we developed a new method to manufacture exoskeletons. For this purpose, we designed and built the HERO (Hand Exoskeleton for Rehabilitation Objectives), an exoskeleton produced using 3D-printed parts embedded with porous fabrics. A new actuator mechanism was also developed in order to reduce costs and test the FDM technique to build functional parts for rehabilitation devices. The HERO was controlled by EEG signals and conceptually tested with one healthy subject undergoing motor MI paradigms, i.e., the imagination of a movement without activating any muscle.

## 2. Methods

### 2.1. Exoskeleton Design

**Figure 1:**
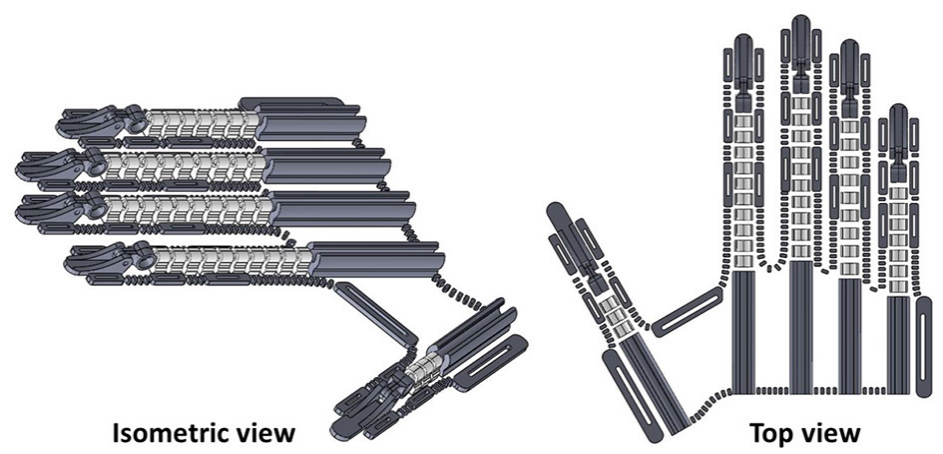
Three-dimensional (3D) design of Hand Exoskeleton for Rehabilitation Objectives (HERO). Isometric view at left and top view at right.

The parts of the HERO were designed and aligned on a Cartesian plane that simulates a 3D printer table. These parts were expected to attach to the fabric during printing and retain the glove format. At first, the dimensions used to align the pieces were specified by the mean size of measurements of 10 male volunteers' hands. Furthermore, end-user hand measurements should be used to customize the exoskeleton. The HERO design is presented in Figure 1. All 3D parts can be found by accessing the following reference: Araujo (2021).

#### 2.1.1. Manufacture Tests

FDM technique with PLA filament was used to prototype the hand exoskeleton. PLA is a biodegradable polymer composed of polylactic acid molecules whose manufacturing process uses renewable materials, such as carbohydrates from corn, potatoes, or dairy products (Auras et al., 2004). Besides, PLA filaments result in prints with better finishing and geometric tolerances (Cal̀ı et al., 2012).

**Figure 2:**
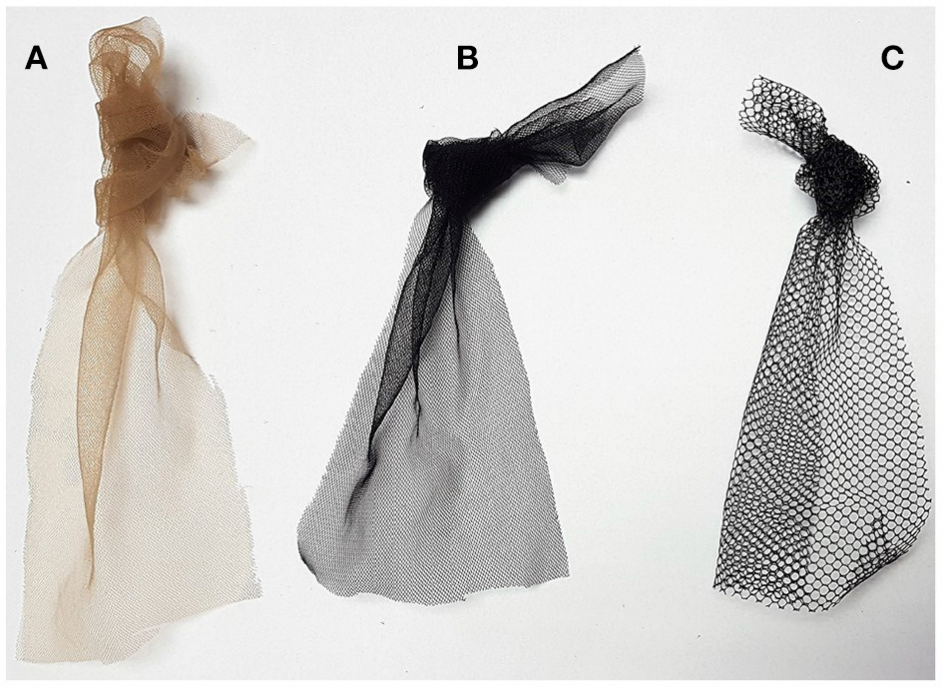
Fabric samples. **(A)** T1 sample. **(B)** T2 sample. **(C)** T3 sample.

Three different fabrics were previously tested. All of them are commercially known as tulle, a lightweight, and resistant netting most commonly used to make bridal veils. Figure 2 shows the chosen tulles. They are composed mainly of polyester but vary in structure, color, and elasticity. The T1 and T2 samples have similar mesh openings and thicknesses, although they have different colors. T3 sample was chosen because of its larger hexagonal mesh compared to T1 and T2. T3 is thicker and less elastic than other samples.

**Figure 3:**
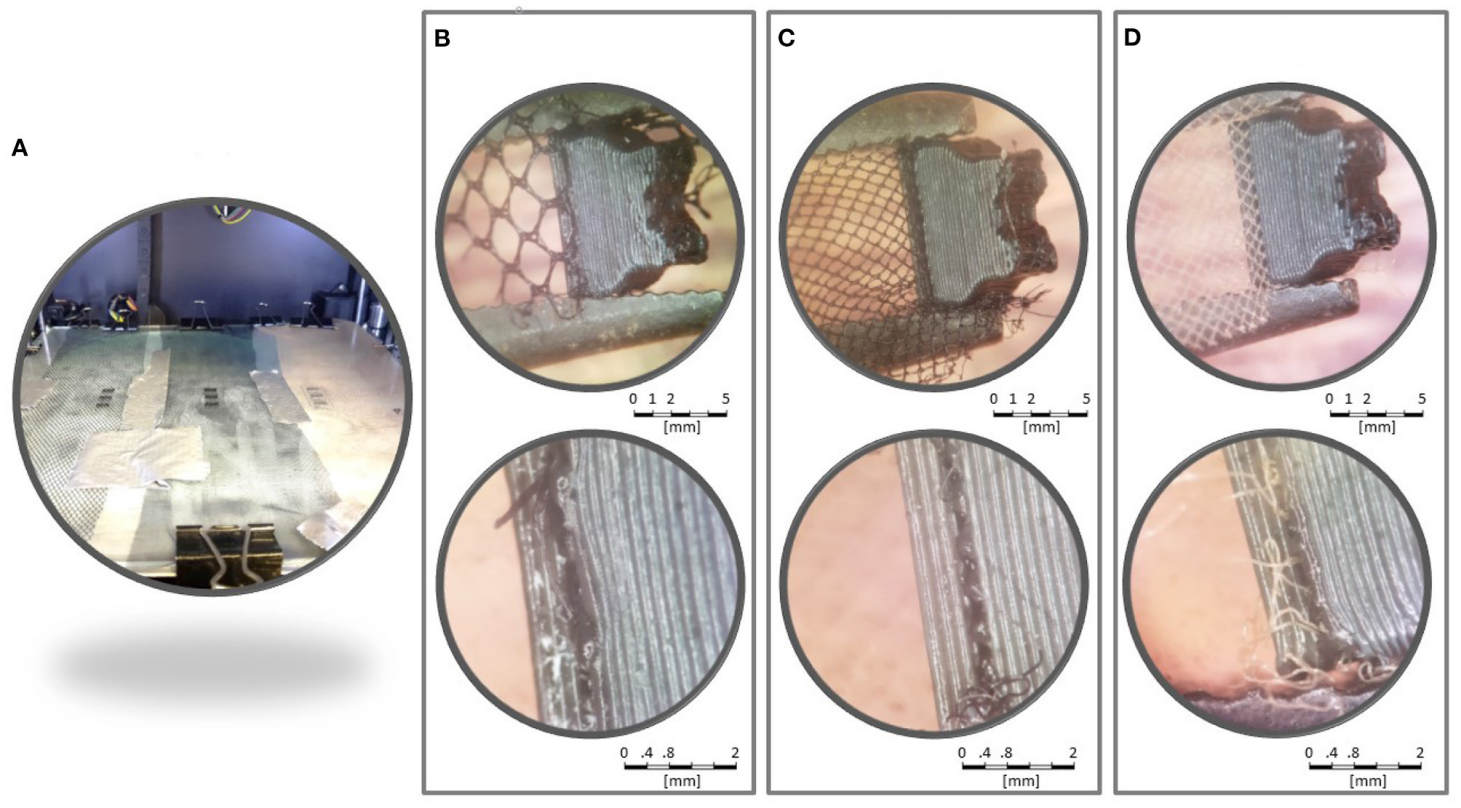
Printing tests. **(A)** All three samples attached to the heated bed. Samples T3 **(B)**, T2 **(C)**, and T1 **(D)** were analyzed in a stereoscopic microscope.

Printing tests were developed in each fabric with some parts of the exoskeleton. Printing parameters were as follows: print speed of 50 mm/s, infill density of 70%, layer high of 0.2 mm, extruder temperature of 211°C, heated bed temperature of 65°C, and no support. G-code was adapted by inserting a pause code M25 followed by the code G1 Z100 to raise the nozzle 100 mm after completing the third layer. After that, the fabric was attached to the table with paper clips and duct tape (see Figure 3A). Then, printing was resumed manually.

Figures 3B–D show amplified pictures of the printing tests. The parts printed were firmly attached to the tulles. PLA layers were observed surrounding fabric fibers. Attempts to separate the parts from the fabric revealed no tendency for delamination between the intersection layers with the fabric. T1 and T2 were similarly strong. Both T1 and T2 seemed more resistant than T3.

#### 2.1.2. 3D Printing

T1 sample was chosen due to its resistance and its color similar to the skin. Based on the test results, two pieces of T1 were inserted between layers 3–4, and layers 6–7 to improve the exoskeleton's mechanical resistance. In this case, the pause code M25 followed by the code G1 Z100 was added after layers 3 and 6. The same printing parameters described in section 2.1.1 were used. See Araujo (2021) to access the .stl file used to print the exoskeleton's glove.

**Figure 4:**
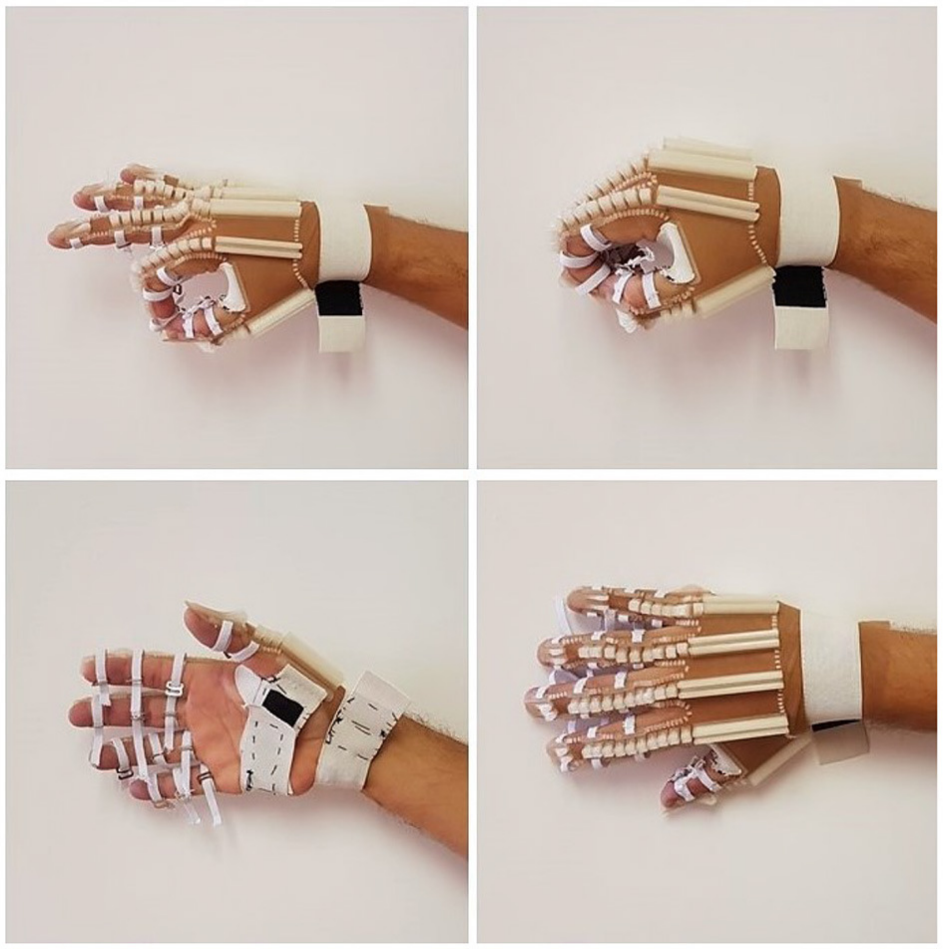
Final prototype of the exoskeleton's glove.

After the printing, scissors were used to cut the fabric along the outline of the fingers. A cut was made up to the wrist region so that an elastic band with hook-and-loop fasteners could be sewn. More of those elastic bands were sewn around the exoskeleton fasteners to attach it to the fingers phalanges and the palm. See Figure 4 for more details.

### 2.2. Actuator Design

**Figure 5:**
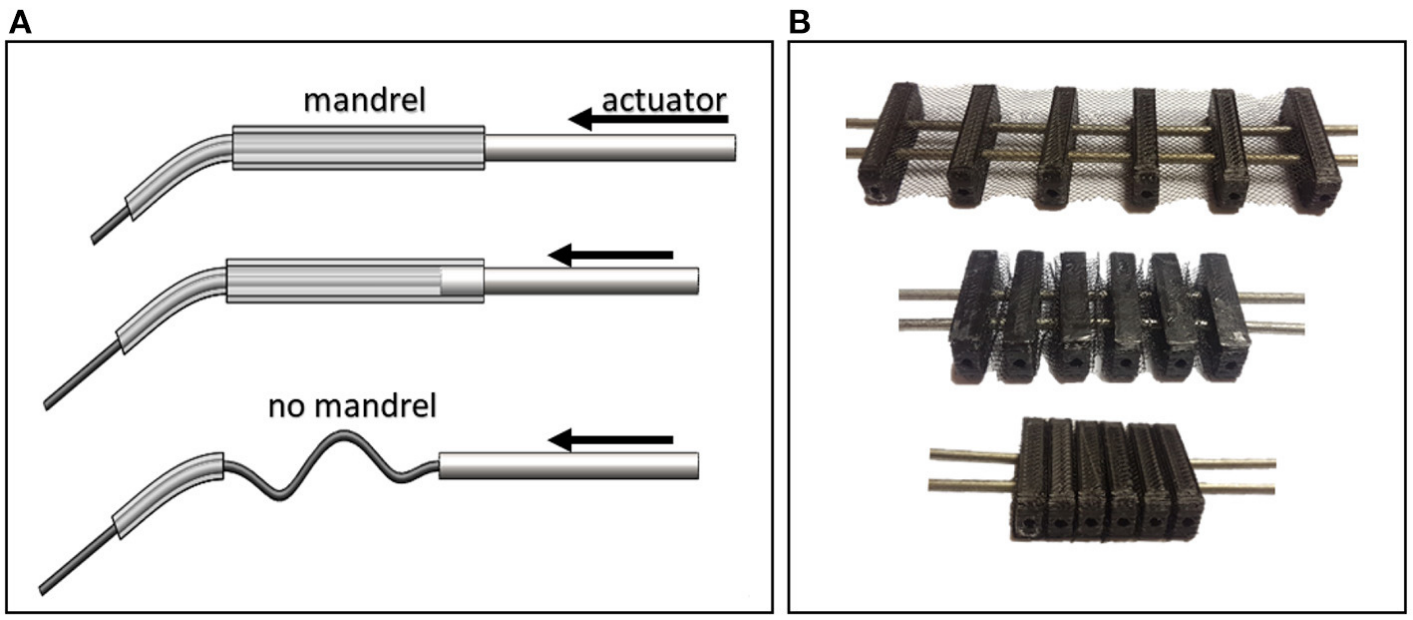
Cable-driven mechanisms. **(A)** The absence of a mandrel to wrap the cable can cause it to flex under compression. This mechanism is not dimensionally efficient by virtue of its long length. **(B)** The Concertina mechanism, which consists of six limiters to prevent cables from bending.

HERO actuation system was developed in order to reduce costs and to test the potential of the FDM technique to build functional parts, but other commercial solutions could be used. The actuator was designed to be attached to the leg. A cable-driven system was chosen to favor device portability and lower complexities. These systems commonly use linear servo motors to pull and push Bowden cables. However, this configuration requires a mechanism similar to the described in Figure 5A to keep the cable always restricted to a wrapped area preventing non-desired bending under compression. Thus, if an actuator stroke of *x* mm is required, one would also need a *x* mm mandrel resulting in a final length of 2*x* mm, not dimensionally efficient.

**Figure 6:**
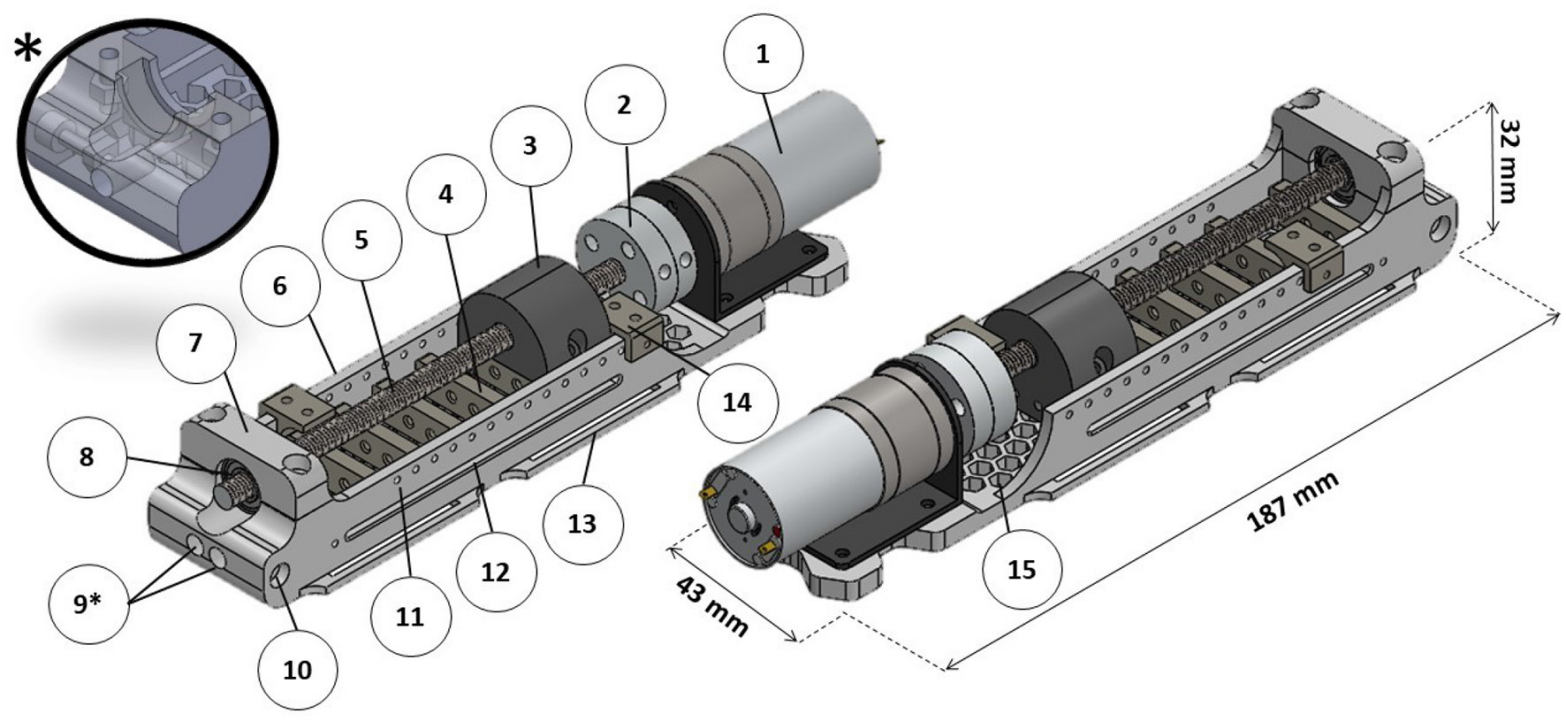
Actuator three-dimensional (3D) model. (1) DC motor; (2) universal couplings for 6 mm (for the lead screw) and 4 mm (for the DC motor); (3) main nut; (4) concertina mechanism; (5) stainless steel M6x1 lead screw; (6) actuator base; (7) bearing mandrel; (8) ball bearing; (9) cable housing entries (*auxiliary view of a model adaptation for use with only one cable); (10) cable housing fixation screw; (11) pin holes to fix micro-switch bases; (12) main nut pins groove; (13) fixation bracket; (14) micro-switch base; (15) honeycomb structure.

Thus, a new actuator was developed using a concertina-like mechanism to drive the HERO movements with the same manufacturing methodology as the exoskeleton (see Figure 5B). This mechanism limits a maximum gap where the cable can pass through under compression without flexing. Figure 6 shows the actuator 3D assembly model.

Each actuator is capable of moving two cables at the same time. The maximum stroke is 60 mm. To move all five fingers, two actuators were required. An extra actuator was designed with one cable to move only the thumb. The actuator has a principle similar to ordinary linear actuators in which a lead screw driven by a DC motor moves a nut to apply force to pull or to push the cables according to the rotation direction. All nuts required for the project were inserted during 3D printing. A honeycomb structure was applied to the actuator base to reduce weight. All 3D parts, as well as the list of extra materials, can be found by accessing the following reference: Araujo (2021).

#### 2.2.1. Torque Requirements

Typical ADLs, such as manipulating small objects with pinch and palmar grips, usually require forces up to 20 N at the fingertips (Lambercy et al., 2007). Studies regarding post-stroke pathological conditions of the hand have shown that torques of 0.9 N · m applied to the metacarpophalangeal joint are enough to extend the four fingers (except thumb) of patients with severe spasticity in flexor muscles (Kamper and Rymer, 2000; Kamper et al., 2006). Therefore, it was established that a criterion of at least a 0.25 N · m torque per finger should be applied to the metacarpophalangeal joint.

**Figure 7:**
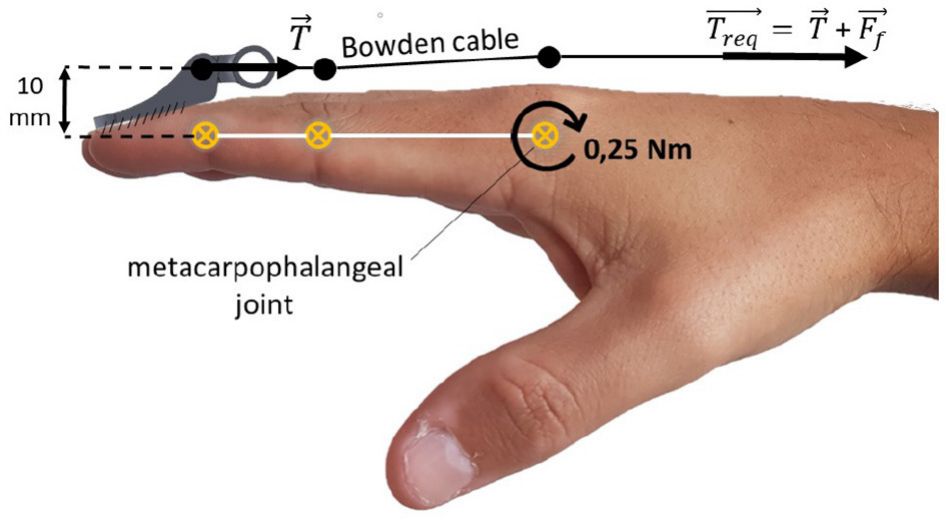
Static scheme for cable tension estimation. *T* is the estimated cable tension, *T*_req_ is the required cable tension, and *F*_f_ is the Bowden cable friction force.

Considering the finger as a rigid body and a lever arm equal to 10 mm between the cable attachment point and the joint (as indicated in Figure 7), it is assumed that the momentum generated by the cable tension should be equal to the momentum applied to the joint. This leads to a *T* tension of 25N.

However, friction also needs to be considered as it influences directly Bowden cables' efficiency. Friction depends on materials of both cable and cable housing as well as on the curvature angle between the actuator and the fixation point (Goiriena et al., 2009). According to Carlson et al. (1995), the required tension must be calculated by Equation (1) to account the friction.

(1) $$\begin{array}{r} T_{\text{req}}=Te^{\mu \theta } \end{array}$$

Where *T*_req_ is the actuator required force, μ is the coefficient of dynamic friction between the cable and the cable housing, and θ is the curvature angle of the cable in radians. A 15.0 N friction force was calculated (37.6% of *T*_req_), considering a tension criteria of *T* = 25 N, a coefficient of friction of μ = 0.150—according to Carlson et al. (1995)—and a curvature angle of π radians between the actuator on the leg and the exoskeleton on the hand. Therefore, the required tension per finger was *T*_req_ = 40.0 N.

A required DC motor torque τ_req_ of 0.055 N · m was calculated using Equation (2), considering a M6x1 lead screw lubricated with machine oil (Budynas and Nisbett, 2011).

(2) $$\begin{array}{r} \tau_{\text{req}}=\frac{Fd_{\text{m}}}{2}\left(\frac{l+\pi fd_{\text{m}}\sec\alpha }{\pi d_{\text{m}}-fl\sec\alpha }\right) \end{array}$$

where *F* is the axial force, i.e., the same as *T*_req_, *d*_m_ is the pitch diameter of the threaded bar, *l* is the thread pitch, *f* is the coefficient of dynamic friction between the nut and the bar, and α is half the thread angle.

#### 2.2.2. Electrical and Power Configurations

Based on the torque requirements, a Ningbo Leison (Ningbo, China) DC motor with a reduction box was chosen. An Arduino Uno and an Arduino shield VNH2SP30 (for more information see Sparkfun, 2014) full-bridge motor driver controlled DC motors rotation. The Arduino code can be accessed at: github.com/rommelaraujo/HERO_actuator_control. Part of the code was dedicated to resetting the actuator to the initial position, i.e., extended fingers, each time the reset button was pressed.

Microswitches were coupled to the actuator base to indicate stroke limits. The actuator bases could be re-positioned, changing the actuation maximum stroke. This acted as a safety mechanism to avoid excessive flexion or extension.

A 12 V sealed lead-acid battery with 5 ampere hours was dedicated to power the actuators' electric system. Although this is a heavy choice, the battery was not coupled to the human body. Alternatively, lithium-ion batteries could also be used in this case.

#### 2.2.3. 3D Printing

Actuator parts had the same printing parameters: print speed of 50 mm/s, infill density of 50%, tri-hexagon infill pattern, layer high of 0.2 mm, extruder temperature of 211°C, and heated bed temperature of 65°C. The only exception was the infill density of 100% to print the microswitch base (part 14 in Figure 6).

The actuator base was printed using support everywhere. Printing was paused after layer 58 to insert two M2.5 nuts used to fix the cable housings and after layer 79 to insert two M3 nuts. The main nut was printed with no support. Printing was paused after layers 43 and 85 to insert two M6x1 lead screw nuts, respectively, and after layer 62 to insert two M2.5 nuts. The concertina mechanism was printed with no support. Printing was paused after layers 6 and 36, respectively, to insert the fabric.

#### 2.2.4. Actuators Mechanical Assessment

Tests were performed to access actuators' mechanical capacity. The cable was coupled to a load cell that transmitted force data to an Arduino Uno. The DC motor was supplied with voltages ranging from 3 to 12 V in steps of 1 V. The maximum pull force was measured three times for each voltage.

### 2.3. HERO Assembly Instruction

HERO assembly instructions can be found at Araujo (2021), including specifications on fixing and coupling parts.

### 2.4. BMI Control Setting

A motor imagery EEG-based BMI was implemented to control the HERO movements using the Graz-BCI paradigm (Pfurtscheller and Neuper, 2001). This paradigm is widely used in human–computer interaction through MI training. OpenVibe (OV) scenarios, an open-source software, were used to extract features from the brain signals and to decide between different MI tasks. These scenarios use a common spatial pattern (CSP) filter plus a linear discriminant analysis (LDA) classifier (Renard et al., 2010). This process consists of the following steps: EEG signal monitoring, EEG signal acquisition, training section with CSP filter, LDA classifier trainer, online feedback, and replay. See OpenVibe (2011) and Silva et al. (2019) for more OV paradigm details.

**Figure 8:**
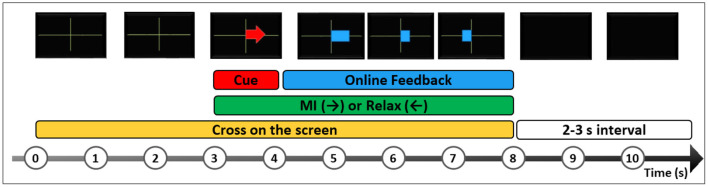
A trial timeline representation of Graz-BCI paradigm on OpenVibe (OV). The participant is instructed to look at a black screen and wait for a green cross, which indicates the beginning of the trial. After 3 s, a red arrow cue appears randomly to the right or to the left. The left arrow indicates the task to relax (C1), and the right arrow indicates the task to perform right hand MI (C2), without any hand movement. After 1 s, a blue feedback bar appears to indicate MI performance in real-time during 4 s followed by 2–3 s to start the next trial. A BMI session has at least 20 trials for each class.

After a training section, the CSP plus LDA classifier had to detect two classes while offering online current BMI activation as feedback in a blue bar. In class 1 (C1), a left arrow appeared on the screen: the volunteer was instructed to relax. In class 2 (C2), a right arrow appeared on the screen: the volunteer was asked to perform a continuous right hand MI movement but keeping his right hand relaxed. No suggestions for MI hand movements were made so that the volunteer would be free to imagine. Figure 8 shows the timeline for one trial.

To control the HERO movements, the participant underwent one session consisting of at least 20 trials for each class. Whenever the classifier detected a class, a specific stimulus was triggered in OV. The scenarios were customized by adding a plugin to communicate with the external actuator control application. A Virtual Reality Peripheral Network server (Button VRPN server plugin) shared data with external devices by exposing boolean virtual button states (on/off). Three buttons were configured in the box. The first or the second buttons turned on whenever a C1 or C2 occurred, respectively. Button 3 only turned on to indicate the beginning of a new trial.

The VRPN library was compiled in Microsoft Visual Studio 2013® (MVS 2013). A C++ program was developed to ensure that OV buttons output data were transferred via USB port to the Arduino Uno. Therefore, C++ program sent values “0,” “1,” or “2” to the Arduino UNO whenever buttons 1, 2, or 3 were turned on, respectively. To access the codes and program details (please see Silva et al., 2019).

A command to push the cables ran each time the Arduino Uno received the value “1” (button 2 ON), passively flexing the fingers as long as button 2 remained ON. Motors were braked if the value “0” (button 1 ON) was detected. At the beginning of each new trial, the Arduino received the value “2” (button 3 ON) and repositioned the actuator to the beginning of the stroke.

### 2.5. Proof of Concept

A proof of concept was developed to test HERO feasibility due to its novel design and approach. The goal was not to compare participant BMI classifier performance since we used previously already tested BMI scenarios. For detailed information about BMI training, please see the previous studies (Ramos-Murguialday et al., 2013, 2019; Donati et al., 2016).

One right-handed healthy male volunteer was invited to use the HERO after the approval of an Ethics Committee of the Universidade Potiguar (No. 79649717.0.0000.529). The participant, naive to BMI training, underwent a MI training session, and an experimental session with online feedback. Each session consisted of a total of 20 trials of C1 and 20 trials of C2 randomly assigned.

#### 2.5.1. EEG Acquisition

Brain signal was acquired with 16 active electrodes (ActiCAP® Brain Products GmbH, Germany), and a V-AMP® amplifier (sampling rate 512 Hz, Brain Products GmbH, Germany). Electrodes were positioned in FC3, C5, C3, C1, CP3, P3, Fz, Cz, FC4, C2, C4, C4, CP4, P4, CPz, and Pz according to the international 10-10 system. The reference was positioned at FCz, and the ground was at AFz. The following requirements were established for signal acquisition and processing: impedance less than 10 kΩ, Butterworth band-pass filtering between 8 and 30 Hz, epoch duration of 4 s with an offset of 0.5 s, and BMI classification accuracy higher than 70%.

#### 2.5.2. Ergonomic Evaluation

Natural grasp movements of the subject were compared to the passive movements developed by HERO. The fingers trajectories were calculated by tracking a white dot positioned on the index fingertip with the Kinovea software (kinovea.org).

Pearson's correlation coefficients (r) were calculated to evaluate the statistical relationship between x and y trajectories for each movement (natural grasp and passive movement). A confidence interval of 5% was chosen. Concordance Correlation Coefficients (CCC) (Lawrence and Lin, 1989) were also calculated to evaluate reproducibility between trajectories.

## 3. Results

Figure 4 shows the HERO prototype printed with translucent PLA and T1 fabric after cutting the fabric and adding fixing elastics. This configuration achieved the best esthetic design. The glove material-only costs were less than US$ 1 (see Supplementary Table 1).

Three actuators were responsible for flexing/extending all five fingers. A two-wire actuator was configured with a 50 mm stroke to drive the index and little fingers. Another two-wire actuator was configured with a 60 mm stroke to drive the middle and ring fingers. Finally, a 40 mm stroke one-wire actuator was responsible for driving the thumb. Each actuator weighed about 240g.

**Figure 9:**
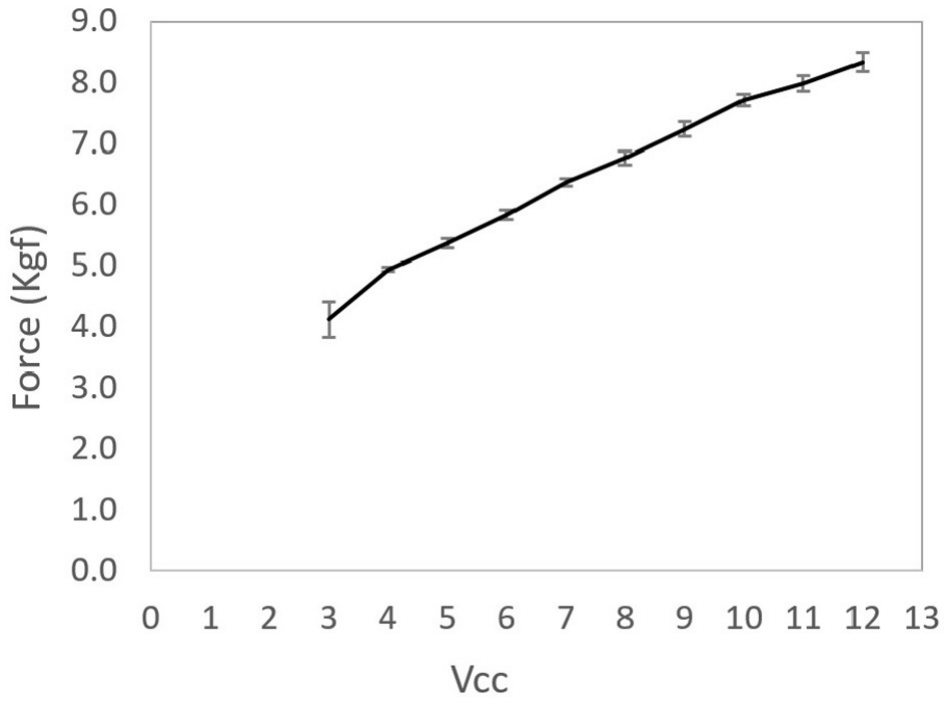
Average of the actuating force per actuator voltage supply. Error bars presented in the figure represent the standard deviations of each mean.

The mean force (in kgf) curve is presented in Figure 9 with their respective standard deviations. The relationship between the maximum actuator force and the voltage supply presented a linear behavior. The peak value was 8.35 kgf, equivalent to 81.9 N when supplied with 12V.

### 3.1. HERO Assembly

**Figure 10:**
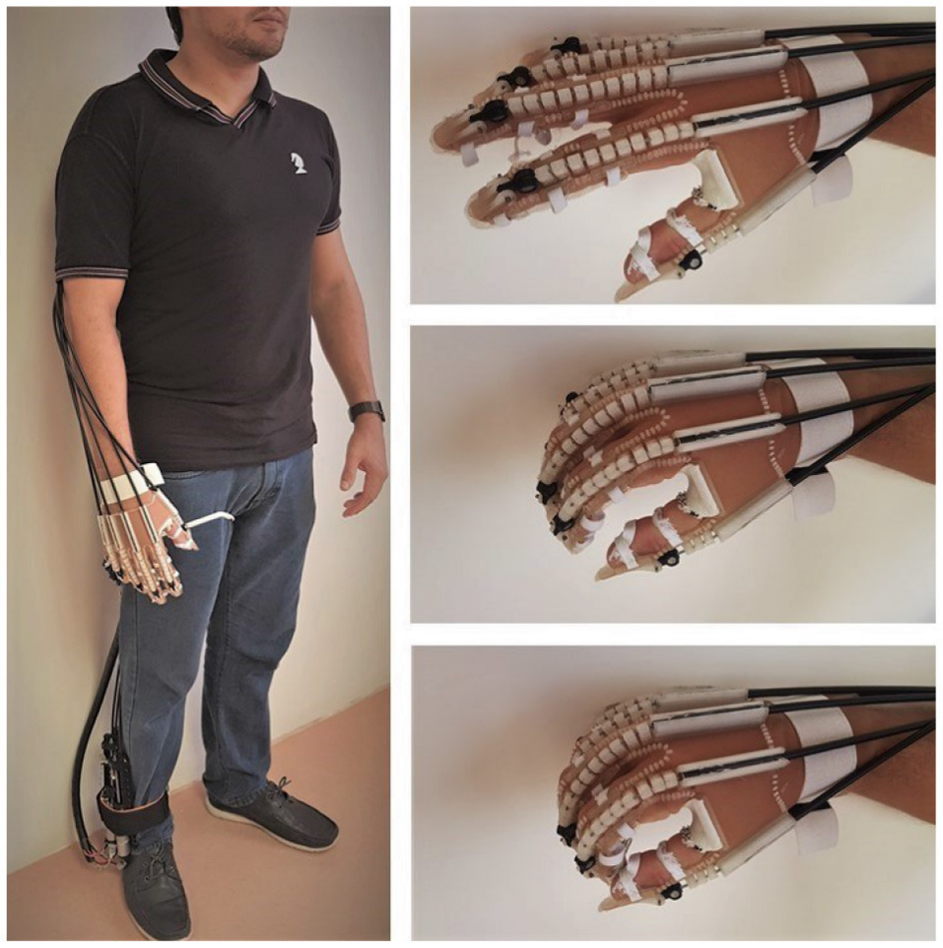
The Hand Exoskeleton for Rehabilitation Objectives (HERO) attached to the body. The actuators were attached to the right lower limb, on the same side of the exoskeleton glove, only for illustration. Patients should have the actuators attached to their healthy lower limb.

The exoskeleton and the actuator were connected by inexpensive bicycle brake cables and cable housings. Figure 10 shows HERO assembled to the body. Actuators were fixed to the leg and the cable housings were hidden by the user's clothes. Elastic bands were attached to the forearm to hold the cables and to give greater mobility. The exoskeleton weighed around 102 g after mounted over the hand, which corresponds to only 20% of the maximum weight suggested by Aubin et al. (2013). The weight of the control system plus the actuators was less than half of the maximum weight requirement (Polygerinos et al., 2015), or, more specifically, 1.4 kg—588 g of battery, 129 g of the Arduino Uno, motor shield, and connection cables, and 683 g of actuators.

### 3.2. Proof of Concept

#### 3.2.1. Control Performances

**Figure 11:**
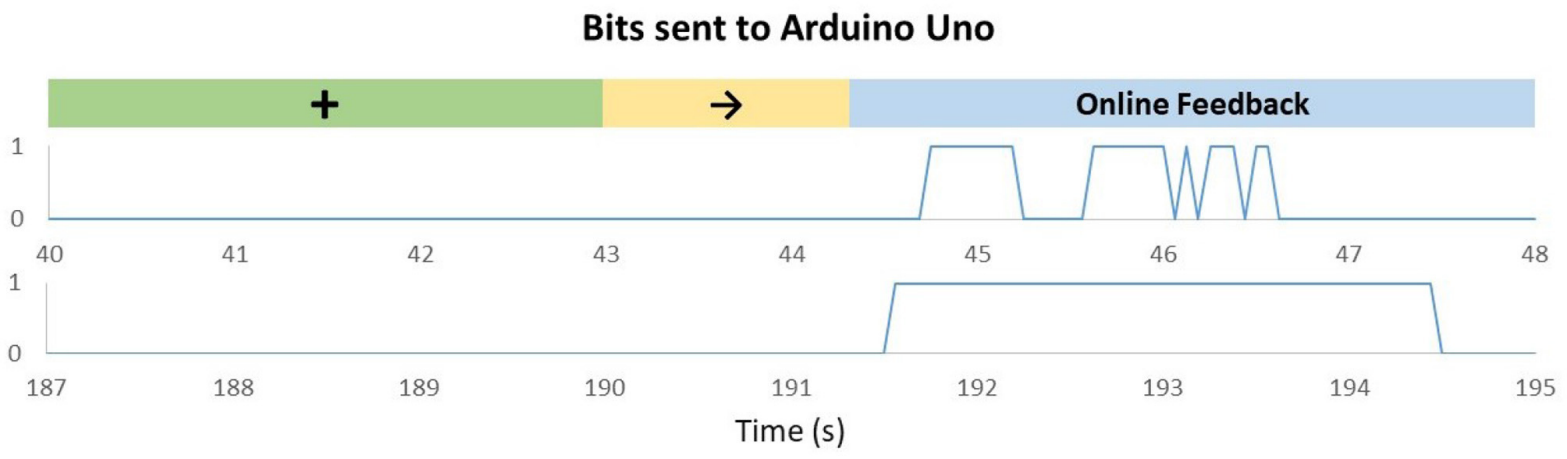
Bits sent to Arduino Uno according to OpenVibe (OV) algorithm outputs in trials with a right arrow [motor imagery (MI) of the right hand]. The green mark represents the moment in which the cross appears on the screen. The yellow mark represents the moment in which the cue to class 2 appears on the screen. The value “0” means the actuators are braked, and the value “1” means the actuators are activated. Upper graph: Trial recorded between 40 and 48 s showing that the feedback can work in a non-continuous mode, according to the decoding received from the MI. Bottom graphic: Trial recorded between 187 and 195 s.

The naive subject was able to control the HERO with a classification accuracy of 91.5%. The variation between trials directly influenced the HERO control. Trials in which the algorithm was unable to accurately predict the correct class led to intermittent passive movements. This situation can be observed in Figure 11. This figure shows the values sent to the Arduino Uno in different trials. HERO actuators were activated only when the Arduino received the value “1.”

The upper graph represents a low-performance trial. In this case, the actuators were activated for short periods, and the exoskeleton was not able to reach the maximum movement range at the end of the trial. In trials with higher performances—lower graph—the actuators had enough time to reach their maximum stroke, and, therefore, full flexion was achieved.

#### 3.2.2. Ergonomic Evaluation

**Figure 12:**
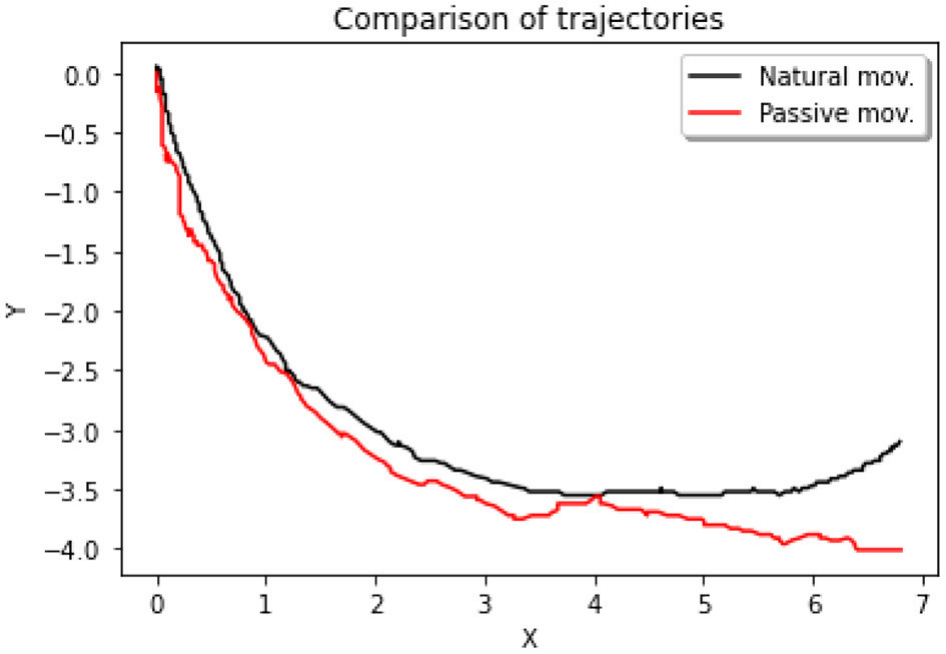
Two-dimensional (2D) trajectories of finger flexion movements. The black curve represents the trajectory of the index fingertip during the execution of an active/natural movement. The red curve represents the passive movement.

Figure 12 describes the trajectory of the index fingertip during finger flexion for natural (actively performed by the subject) and passive movements (performed by the exoskeleton).

**Figure 13:**
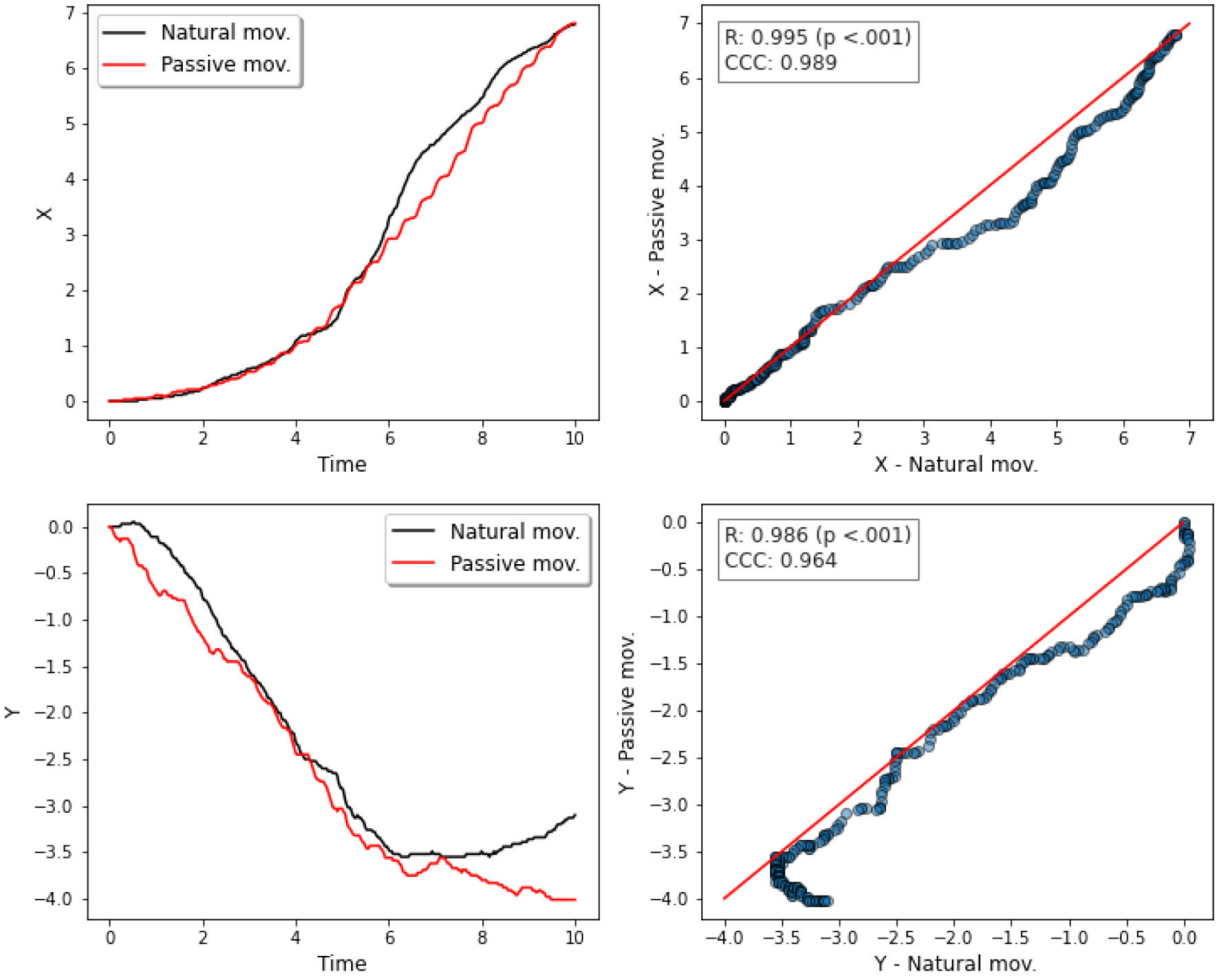
Correlation analyses between x and y trajectories. First column: x and y trajectories vs. time for natural and passive movements. Second column: scatter plots with the 45° line (in red) to compare both movements—natural and passive—for x (top) and for y (bottom) trajectories.

There was a positive correlation between the natural movement on the x axis, and the passive movement on the x axis, Person's *r* = 0.995, *p* < 0.001, *N* = 302 (see Figure 13). We also observed a positive correlation between the natural movement on the y axis, and the passive movement on the y axis, Person's *r* = 0.986, *p* < 0.001, *N* = 302.

Reproducibility analysis indicated positive correlation for both movements in both trajectories, *CCC* = 0.986 (x axis), and *CCC* = 0.964 (y axis).

## 4. Discussion

### 4.1. Manufacturing Methodology

In this work, for the first time, an innovative manufacturing method was applied within the scope of assistive technologies and Soft Robotics devices to make a low-cost and lightweight hand exoskeleton for stroke rehabilitation. The fabric-associated FDM technique provided rapid and versatile HERO prototyping.

HERO prototype was designed aiming to be discreet and esthetically pleasing, although it has not been evaluated for final consumers yet. The combination of the transparent PLA with the invisible tulle gives the impression that the exoskeleton makes part of the hand. This esthetic aspect is one of the key features to consider when designing robotic devices for rehabilitation (McConnell et al., 2017). The HERO low-cost design may contribute to better acceptance by stroke patients and may motivate intensive hand rehabilitation training and possible use during ADLs.

FDM technique had several advantages, such as good esthetic finishing, precision in dimensional tolerances, rapid and versatile prototyping, and easy replacement of parts. The counterpoints were as follows: the necessity of constant printer maintenance to guarantee high-quality parts and uncertainties about using this methodology to mass production.

HERO design used dimensional tolerances of a 0.3 mm gap for clearance fits and a 0.15 mm gap for transition fits. These values are in line with previous work (Cal̀ı et al., 2012). However, factors such as incorrect table calibration, ambient temperature, and extruder temperature variations directly affect the quality of the joints. Nevertheless, this work showed that functional parts can be produced by the FDM technique, contributing to the findings of previous works (Zuniga et al., 2015; Fix-it, 2019).

### 4.2. Durability

The “fabric sandwich” durability over intensive use is still unknown. The actuator also needs to be evaluated in this regard. Therefore, further extensive durability tests are necessary. Design changes such as part resizing, different fabric configurations, and also the application of seams could increase durability.

### 4.3. Mechanical Specifications of Actuators

In this study, a torque criterion of 0.25 N · m was adopted based on previous works (Kamper and Rymer, 2000; Kamper et al., 2006). According to these studies, the specified torque is enough to use the HERO in ADLs and in clinical procedures with spastic stroke patients. This criterion was higher than those generally adopted in exoskeleton projects (Arata et al., 2013; Randazzo et al., 2018). However, a thorough analysis still needs to be done to assess the real force applied to the fingertips and to overcome spasticity in stroke patients.

### 4.4. Weight

The weight of the hand-mounted exoskeleton increased from 58 to about 102 g by adding cables and cable housings. Nevertheless, it is only about 20% of the 500 g maximum criterion suggested by Aubin et al. (2013). It is also below the average weight of cable-driven exoskeletons (Chu and Patterson, 2018). The control system weighed about 1.4 kg. Although this value is within the initial specification of 3 kg (Polygerinos et al., 2015), many adjustments still can be done such as the use of lighter batteries. It is noteworthy that the mentioned values do not consider the BMI apparatus.

### 4.5. Cost

The material-only costs were equivalent to US$ 125.00. This is an inexpensive value considering the potential for rehabilitation intervention that could be applied intensively in post-stroke patients. We believe that the values can be reduced even further in future versions.

### 4.6. Ergonomics

Passive motion performed by the HERO was compared to the natural hand-grip movement. The analysis showed a strong positive correlation between movements in x and y positions. Reproducibility analysis also indicated substantial concordance in both cases.

Although the HERO mechanical system is not able to perform complex movements, Balasubramanian et al. (2010) suggests that increasing the DOF may not be the best alternative for post-stroke rehabilitation. One of the reasons is that most patients need to recover simple movements before performing more precise movements. In addition, more complex devices involve equally complex control techniques, which may reduce patient enrollment.

### 4.7. EEG-Based BMI

EEG is an interesting alternative for total limb paralyzed stroke patients. However, robust EEG equipment is expensive. Although low-cost devices such as Emotiv Epoc are already available, the signal quality of these devices is questionable, and the use is now based on rental (McConnell et al., 2017).

Alternatively, there are cheaper EEG devices such as the open-source OpenBCI Cyton Biosensing Board (OpenBCI, 2021) or do-it-yourself (DIY) devices where users can build and test their own EEG (OpenEEG, 2019). These devices have already been used in BMI applications and could be associated with HERO (Suryotrisongko and Samopa, 2015; Frey, 2016). However, despite the efforts of DIY EEG designers, aspects such as safety, durability, and reliability are still questionable and not properly specified. It is important to notice that the HERO exoskeleton could work with any EEG device whose electrodes could be positioned over the M1 area, around the electrode C3/C4.

The classification accuracy of the BMI was high for a naive subject, and it can increase with regular training. This high decoding control rate could be interesting for patient rehabilitation, even though it is not related to the HERO design and functionality. Although we have not analyzed the closed-loop latency (i.e., the delay between the algorithm classification, the start of the movements performed by the HERO, and the somatosensory cortex perceiving and processing), some studies (Pfurtscheller and Solis-Escalante, 2009; Müller-Putz et al., 2010) suggest that SMR-based BMI can operate with latencies in the order of seconds.

Balasubramanian et al. (2018) suggests that most stroke survivors are still able to control devices by residual myoelectric activity. Thus, it is possible that human–machine interfaces (HMIs) such as electromyography (EMG) could be associated with the HERO for motor rehabilitation, possibly improving accuracy, and performance (Ribeiro et al., 2013). This would considerably decrease the total costs involved in rehabilitation interventions and facilitate both clinical and intensive use in patients' homes.

### 4.8. Safety

Only two safety mechanisms were implemented: stroke limiters to avoid excessive flexion or extension and reset button. Other safety mechanisms such as emergency buttons and band sensors suggested by Chu and Patterson (2018) will be implemented further.

### 4.9. Limitations

Although the required momentum to move the fingers of patients with severe spasticity was taken into account, only a proof of concept was performed with a healthy subject. No tests have been performed on stroke patients to assess the usability and acceptability of the HERO. Neither can be inferred whether the HERO EEG-based training can bring clinical benefits to chronic stroke patients such as happened in Ramos-Murguialday et al. (2013). Future clinical studies may provide appropriate answers to these questions. Besides, safety mechanisms have yet to be implemented.

## 5. Conclusion

The development of robotic devices for post-stroke hand rehabilitation has made rapid progress, especially over the last 10 years. Although the neurophysiological basis behind motor recovery is not fully comprehended, few weeks of BMI therapy improves significantly motor control.

Here, we presented an innovative manufacturing technique for developing a low-cost robotic device for hand rehabilitation after stroke. The mechanical design was based on a large literature review. HERO project focused on balancing different parameters, such as esthetics, comfort, practicality, ergonomics, portability, and low cost.

The manufacturing methodology that combines textiles with 3D-printed parts may be an alternative to develop new wearable devices for healthcare. Besides, simple and low-cost parts were used to build the HERO electrical and mechanical systems guaranteeing easy maintenance. The result was a lightweight, simple, portable, and inexpensive device.

Large-scale tests still need to be developed. We expect that HERO training sessions can be performed not only in hospitals and clinical settings but also in patients' homes enabling intensive rehabilitation training and higher movement recovery for stroke patients—or patients with movement disorders. Another possibility is to use the HERO to assist patients in various ADLs, which may increase the quality of life of stroke patients and their caregivers.

## Data Availability Statement

The datasets presented in this article are not readily available, because the data are confidential. Requests to access the datasets should be directed to Rommel S. Araujo, a.rommelsoares@gmail.com.

## Ethics Statement

The studies involving human participants were reviewed and approved by Universidade Potiguar (No. 79649717.0.0000.529). The patients/participants provided their written informed consent to participate in this study.

## Author Contributions

RA conceived of the present idea, design, analytic calculation, data collection and analysis, study concept and design, and wrote the manuscript with support of FB. CS contributed with the HERO serial communication with the Arduino Uno and assisted with other technical details. SN enabled access to the 3D printers from the Laboratory of Technological Innovation in Health (LAIS), assisted with technical details, and revision of the final manuscript. EM supervised the project and helped with material acquisition. FB contributed to the study concept and design, critical revision of the manuscript for important intellectual content, obtained funding, and supervised the project. All authors contributed to the article and approved the submitted version.

## Conflict of Interest

The authors declare that the research was conducted in the absence of any commercial or financial relationships that could be construed as a potential conflict of interest.
