# Supplementary material for: Development of a Low-Cost EEG-Controlled Hand Exoskeleton 3D Printed on Textiles
Source: Front Neurosci. 2021 Jun 25;15:661569. doi: 10.3389/fnins.2021.661569 (PMC8267155; doi:10.3389/fnins.2021.661569)
Supplement: Supplementary file 1 [file Data_Sheet_1.PDF]

frontiers

Supplementary Material

LIST OF MATERIAL COSTS

HERO's glove			
**Part**	**Quantity**	**Total Price (R$)**	**Total Price (US$)***
3D printing filament	31.6 g	R$ 3.63	$ 0.73
Fabric	150 cm²	R$ 0.29	$ 0.06
**Total material cost per glove**		R$ 3.92	$ 0.78
**HERO's actuator set ****			
**Part**	**Quantity**	**Total Price (R$)**	**Total Price (US$)***
3D printing filament	257.9 g	R$ 29.66	$ 5.93
Bowden cables	5	R$ 10.00	$ 2.00
DC motors	3	R$ 177.00	$ 35.40
Microswitches	6	R$ 2.40	$ 0.48
Monster Motor Shield VNH2SP30	2	R$ 107.00	$ 21.40
Arduino Uno	2	R$ 79.80	$ 15.96
Flexible couplers	6	R$ 72.00	$ 14.40
Fastening components	-	R$ 20.00	$ 4.00
Sealed lead-acid battery 12V	1	R$ 119.90	$ 23.98
**Total material cost per actuators set**		R$ 617.76	$ 123.55
**Total material cost**		R$ 621.67	$ 124.33

Table S1. List of materials and related costs to build the HERO.
* Considering the quotation of US$ 1 (one American dollar) equal to R$ 5.00 (five reais)
** Data equivalent to 3 actuator units
